# Clinical characteristics of children with septic arthritis caused by different pathogenic bacteria

**DOI:** 10.1002/pdi3.2522

**Published:** 2025-01-10

**Authors:** Ziyu Li, Gefang Li, Jun Wu, Bo He, Qun Zhang

**Affiliations:** ^1^ Children's Hospital of Chongqing Medical University Chongqing China; ^2^ Department of Pediatric Orthopedics Children's Hospital of Chongqing Medical University, National Clinical Research Center for Child Health and Disorders, Ministry of Education Key Laboratory of Child Development and Disorders, Chongqing Key Laboratory of Pediatrics Chongqing China

**Keywords:** children, clinical manifestations, pathogenesis, retrospective analysis, septic arthritis

## Abstract

Septic arthritis is a serious infectious disease in children. In this study, we retrospectively analyzed the relationship between demographics, laboratory values at presentation, reported symptoms at presentation, joint involvement, and distribution characteristics of pathogenic bacteria in 171 patients during the period of 2012–2022. The results showed that a total of 77 pathogen‐positive patients were detected in the 171 patients (culture‐positive rate of 45.0%), 15 categories of pathogenic bacteria, and the highest detection rates were 52 cases of *Staphylococcus aureus* (67.5%), *Streptococcus pyogenes* in 7 cases (9.1%), and *Streptococcus pneumoniae* in 5 cases (6.5%). Comparison of these three pathogens revealed that the age of the *Staphylococcus aureus* and *Streptococcus pyogenes* groups was significantly older than that of the *Streptococcus pneumoniae* group (*p* < 0.05), and that the white blood cell count (WBC) of the *Streptococcus pyogenes* group was significantly higher than that of the *Staphylococcus aureus* group (*p* < 0.05), and that the *Streptococcus pyogenes* group had a significantly higher procalcitonin (PCT), which was significantly higher in the *Streptococcus pneumoniae* group (*p* < 0.05). The results suggest that the pathogenic bacteria of septic arthritis in children are mainly *Staphylococcus aureus*, *Streptococcus pneumoniae* infection occurred more commonly in the infant stage, and *Staphylococcus aureus* and *Streptococcus pyogenes* were detected frequently in school‐age children. *Streptococcus pyogenes* was found to be more pathogenic than *Staphylococcus aureus* and *Streptococcus pneumoniae*.

## Graphical abstract

Children with septic arthritis infected by *Streptococcus* *pneumoniae* were younger than those by *Staphylococcus aureus* and *Streptococcus* *pyogenes*, and patients with *Streptococcus* *pyogenes* had the highest WBCs and PCTs, The children in the *Streptococcus pneumoniae* group had a significantly longer time until presentation than those in the *Streptococcus pyogenes* group, with statistically significant differences.

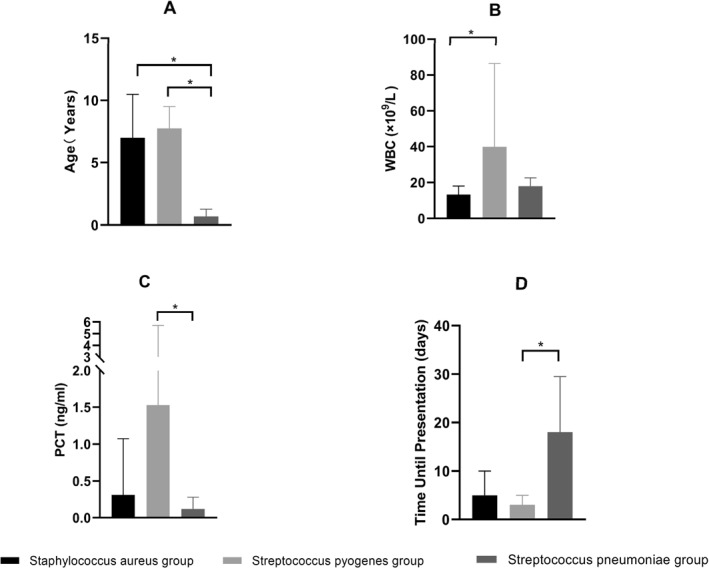

## 1 INTRODUCTION

Septic arthritis (SA) is a joint infection caused by bacteria, viruses, or fungi.^1^ It is most common in children and accounts for about 21% of childhood bone and joint infections,^2^ with an overall incidence of 4–10 cases per 100,000 population.^3^ SA is a critical infectious disease in pediatric orthopedics, with a mortality rate of 11%.^4^ Early identification of the causative organism and effective antibiotic therapy are key to improving patient prognosis and promoting functional recovery. Clinicians often administer empirical broad‐spectrum antibiotics before the microbiological culture results are available, which can increase organism drug resistance and delay patient treatment if targeted antibiotic therapy is not selected. To date, most previous studies on SA reported the distribution and drug resistance of the pathogenic bacteria; however, there is a lack of studies on the characteristics of the causative organisms of SA. Therefore, this study analyzed the clinical characteristics, the distribution of pathogenic bacteria, and the characteristics of different pathogenic bacterial infections in children with SA in our hospital in the past 11 years, aiming to assist clinicians in the early identification of pathogenic bacteria and the selection of effective antimicrobial agents.

## 2 PATIENTS AND METHODS

### 2.1 Patients

For this study, 171 patients with SA admitted to the Children's Hospital of Chongqing Medical University from 2012 to 2022 were selected. This study was reviewed and approved by the Medical Research Ethics Committee of Children's Hospital of Chongqing Medical University, ethical approval number 2022‐343.

#### 2.1.1 Inclusion criteria

We included patients with SA diagnosed by history, clinical manifestations, laboratory tests, and microbiological examination; patients with blood culture or synovial culture results; and patients with a complete medical history and laboratory findings.

#### 2.1.2 Exclusion criteria

We excluded patients with SA combined with a tumor, immunodeficiency, or other inflammatory diseases of the body; patients with SA combined with adjacent osteomyelitis; and patients with a history of trauma or surgery in the adjacent area.

### 2.2 Methodology

#### 2.2.1 Inclusion of observables

The demographic information was collected for all patients including age, and sex. Four inflammatory markers were tested at the time of admission, namely, the white blood cell count (WBC), erythrocyte sedimentation rate (ESR), C‐reactive protein (CRP), and procalcitonin (PCT) levels. Reported symptoms at presentation were noted, including the time until presentation, temperature, joint redness, joint swelling, increased skin temperature, and joint pain. The culture results of venous blood specimens or synovial fluid specimens collected at the time of admission were recorded.

#### 2.2.2 Specimen culture and identification

The collected venous blood and synovial specimens were inoculated on blood agar plates for bacterial culture, and the strains were isolated according to their morphology and Gram staining results. Tests were performed using the Biomérieux VITEK 2 compact fully automatic bacterial identification and drug sensitivity analysis system (Biomérieux, Marcy‐l'Étoile, France).

#### 2.2.3 Statistical analysis

The data were analyzed using SPSS26.0 statistical software (IBM Corp., Armonk, NY, USA). Normally distributed continuous variables were expressed as the mean ± standard deviation ($\bar{x}$ ± s), and an independent sample *t*‐test was used for comparisons between groups. Non‐normally distributed continuous variables were expressed as the median (quartiles, M [P25, P75]), and the Mann–Whitney *U* test and Kruskal–Wallis H (≥ 3 independent groups) test were used for comparisons between groups. Count data were expressed as frequencies and percentages (%), between‐group comparisons were made using the *χ* ^*2*^ test when *n* > 40 and *t* > 5, the continuity‐corrected *χ* ^*2*^ test when *n* > 40 and 1 ≤ *t* < 5, and Fisher's exact probability method for *n* < 40, or *t* < 1. *p* < 0.05 indicated that the differences were statistically significant.

## 3 RESULTS

### 3.1 Comparison of indicators between the blood/synovial fluid culture‐positive and culture‐negative groups

A total of 94 of the 171 patients had negative blood/synovial fluid culture results, with a negative culture rate of 55.0%, and 77 had positive cultures, with a positive culture rate of 45.0%. Patients in the culture‐positive group had significantly higher WBC, CRP, PCT, and temperature than those in the negative group (*p* = 0.009, 0.000, 0.000, and 0.019, respectively). All patients in the culture‐positive group had single joint involvement, with the hip joint being the most frequently involved site (*p* = 0.012). Knee involvement was most common in the culture‐negative group of patients (*p* = 0.011), with only one case showing simultaneous involvement of multiple joints. There was no statistically significant difference between patients in the culture‐positive group and the culture‐negative group in terms of sex, age, ESR level, time until presentation, incidence of joint redness, swelling, increased skin temperature and pain, and involvement of the ankle joints (*p* > 0.05) (Table 1).

**TABLE 1:** Blood/synovial fluid results for the culture‐positive and culture‐negative groups.

Groups		Culture‐positive group (*n* = 77)	Culture‐negative group (*n* = 94)	*p*
Demographics				
Sex (cases)	Male	44	54	0.968
	Female	33	40	
Age (years)		6.75 (1.71, 9.67)	6.21 (3.38, 9.23)	0.677
Laboratory values at presentation				
WBC (×10^9^)		13.32 (8.73, 18.58)	11.02 (8.11, 14.81)	0.009
ESR (mm/h)		80.0 (53.5, 95.5)	63.50 (36.8, 96.0)	0.065
CRP (mg/L)		51.00 (29.50, 86.50)	30.00 (8.00,59.25)	0.000
PCT (ng/mL)		0.320 (0.120,1.020)	0.100 (0.073,0.268)	0.000
Reported symptoms at presentation				
Time until presentation (d)		5.0 (2.5, 11.0)	7.0 (3.7, 8.3)	0.076
Body temperature (°C)		36.8 (36.6, 38.1)	36.8 (36.5, 37.2)	0.019
Reddening of joints (*n* (%))		21 (27.2)	22 (23.4)	0.562
Joint swelling (*n* (%))		34 (44.2)	44 (46.8)	0.729
Increased skin temperature (*n* (%))		43 (55.8)	53 (56.4)	0.944
Joint pain (*n* (%))		56 (72.7)	63 (67.0)	0.420
Joint involvement (*n* (%))				
Hip joint		50 (64.9)	43 (45.7)	0.012
Knee		23 (29.9)	46 (48.9)	0.011
Ankle		4 (5.2)	2 (2.1)	0.439
Elbow joint		0	1 (1.1)	N/A
Sacroiliac joint		0	1 (1.1)	N/A
Hip, knee		0	1 (1.1)	N/A

*Note*: N/A indicates that no statistical analysis was carried out.

### 3.2 Bacterial culture results

Of the 77 patients with positive bacterial culture results Gram‐positive bacteria (G^+^) were detected in 67 cases, Gram‐negative bacteria (G^−^) were found in 5 cases (6.5%), and there were 5 cases (6.5%) of mixed infections. The top three bacteria detected were *Staphylococcus aureus* in 52 cases (67.5%), *Streptococcus pyogenes* in 7 cases (9.1%), and *Streptococcus pneumoniae* in 5 cases (6.5%) (Table 2).

**TABLE 2:** Distribution of culture‐positive pathogenic bacteria in patients with septic arthritis.

	Pathogen	*n*	Composition ratio (%)
Gram‐positive	MSSA	37	48.1
	MRSA	15	19.5
	*Streptococcus pyogenes*	7	9.1
	*Streptococcus pneumoniae*	5	6.5
	*Staphylococcus epidermidis*	1	1.3
	*Staphylococcus haemolyticus*	1	1.3
	*Staphylococcus cohnii urealyticum*	1	1.3
Gram‐negative	*Pseudomonas aeruginosa*	1	1.3
	*Citrobacter fowleri*	1	1.3
	*Klebsiella pneumoniae*	1	1.3
	*Escherichia coli*	1	1.3
	*Salmonella*	1	1.3
Mixed infections	*Acinetobacter baumannii*, MRSA	1	1.3
	Human *Staphylococcus* subspecies, pale green aerococcus	1	1.3
	*Salmonella*, *Staphylococcus epidermidis*	1	1.3
	Human *Staphylococcus*, *Escherichia coli*	1	1.3
	*Streptococcus lactis equi subspecies*, *Staphylococcus aureus*	1	1.3
	Total	77	100

Abbreviations: MRSA, methicillin‐resistant Staphylococcus aureus; MSSA, methicillin‐sensitive Staphylococcus aureus.

### 3.3 Comparison of the indicators in the *Staphylococcus aureus* group, *Streptococcus pyogenes* group, and *Streptococcus pneumoniae* group

**FIGURE 1:**
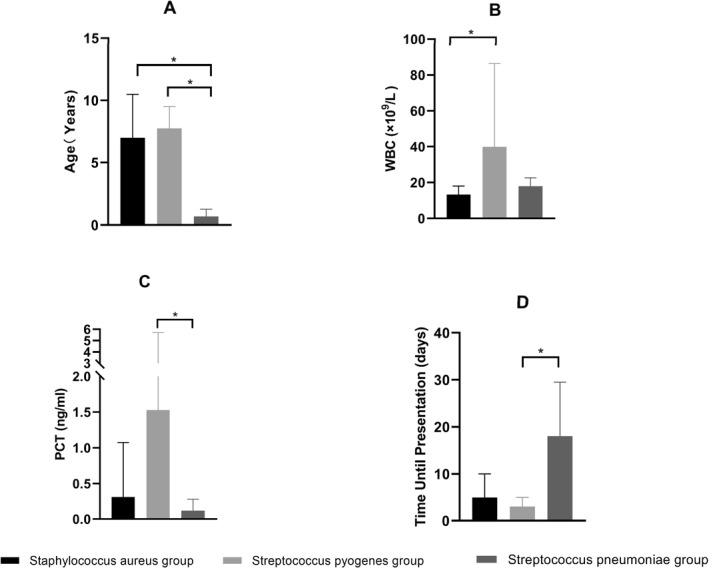
Two‐by‐two comparisons of the *Staphylococcus aureus* group, *Streptococcus pyogenes* group, and *Streptococcus pneumoniae* group in terms of (A) age, (B) WBC, (C) PCT levels, and (D) time until presentation. WBC indicates white blood cell count; PCT, procalcitonin. * Represents *p* < 0.05.

The pathogenic bacteria cultured from the cases included in this study were mainly *Staphylococcus aureus* (*n* = 52), *Streptococcus pyogenes* (*n* = 7), and *Streptococcus pneumoniae* (*n* = 5); therefore, cases of SA were divided into the *Staphylococcus aureus* group, *Streptococcus pyogenes* group, and *Streptococcus pneumoniae* group for statistical analysis. Comparing the indicators of each group revealed that there were statistically significant differences among the *Staphylococcus aureus* group, *Streptococcus pyogenes* group, and *Streptococcus pneumoniae group* in terms of age, WBC, PCT, and time until presentation (*P* = 0.006, 0.001, 0.018, and 0.015, respectively) (Table 3). Further post‐hoc two‐by‐two comparisons using the Bonferroni method to correct for significance levels revealed that the children in the *Staphylococcus aureus* group were significantly older than those in the *Streptococcus pneumonia*e group and the children in the *Streptococcus pyogenes* group were significantly older than those in the *Streptococcus pneumoniae* group. The children in the *Streptococcus pyogenes* group had significantly higher WBC levels than those in the *Staphylococcus aureus* group. The children in the *Streptococcus pyogenes* group had significantly higher PCT levels than those in the *Streptococcus pneumoniae* group. The children in the *Streptococcus pneumoniae* group had a significantly longer time until presentation than those in the *Streptococcus pyogenes* group (Figure 1).

**TABLE 3:** Comparison of the indexes in the *Staphylococcus aureus* group, S*treptococcus pyogenes* group, and *Streptococcus pneumoniae* group.

Group		*Staphylococcus aureus* (*n* = 52)	*Streptococcus pyogenes* (*n* = 7)	*Streptococcus pneumoniae* (*n* = 5)	*p*
Demographics					
Sex (cases)	Male	30	4	2	0.890
	Female	22	3	3	
Age (years)		7.00 (2.48, 10.48)	7.75 (7.25, 9.50)	0.69 (0.46, 1.28)	0.006
Laboratory values at presentation					
WBC (×10^9^)		12.73 (8.81, 16.46)	25.50 (19.14, 30.51)	15.56 (14.17, 23.03)	0.001
ESR (mm/h)		78.00 (56.75, 95.75)	81.00 (43.00, 100.00)	88.00 (48.50, 98.00)	0.876
CRP (mg/L)		57.00 (31.00, 84.25)	124.00 (59.00, 243.00)	35.00 (33.00, 72.50)	0.119
PCT (ng/mL)		0.313 (0.129, 1.074)	1.530 (1.010, 5.707)	0.116 (0.057, 0.282)	0.018
Reported symptoms at presentation					
Time until presentation (d)		5.0 (2.3, 10.0)	3.0 (1.7, 5.0)	18.0 (7.5, 29.5)	0.015
Body temperature (°C)		36.9 (36.6, 38.3)	37.2 (36.6, 38.5)	36.8 (36.4, 36.8)	0.268
Reddening of joints (*n* [%])		15 (28.8)	1 (14.3)	1 (20.0)	0.865
Joint swelling (*n* [%])		19 (36.5)	5 (71.4)	3 (60.0)	0.152
Increased skin temperature (*n* [%])		25 (48.1)	6 (85.7)	2 (40.0)	0.169
Joint pain (*n* [%])		38 (73.1)	6 (85.7)	3 (60.0)	0.655
Joint involvement (*n* [%])					
Hip joint		39 (75.0)	4 (57.1)	2 (40.0)	0.192
Knee		11 (21.2)	2 (28.6)	3 (60.0)	0.142
Ankle		2 (3.8)	1 (14.3)	0	0.470

### 3.4 Comparison of various indicators between the MSSA group and the MRSA group

There was a statistically significant difference between the methicillin‐sensitive Staphylococcus aureus (MSSA) and methicillin‐resistant Staphylococcus aureus (MRSA) groups only for the rate of joint swelling (*P* = 0.027), with no statistically significant differences for the demographic information, laboratory values at presentation, time until presentation, body temperature, incidence of joint redness, increased skin temperature, and joint involvement (all *P* > 0.05) (Table 4).

**TABLE 4:** Comparison of indicators in MSSA and MRSA groups.

Groups		MSSA (*n* = 37)	MRSA (*n* = 15)	*p*
Demographics				
Sex (cases)	Male	23	7	0.306
	Female	14	8	
Age (years)		6.75 (2.20,10.54)	7.83 (4.42,10.42)	0.585
Laboratory values at presentation				
WBC (×10^9^)		13.51 ± 4.90	12.84 ± 4.21	0.640
ESR (mm/h)		77.51 ± 29.36	79.87 ± 24.11	0.785
CRP (mg/L)		59.00 (31.50,94.42)	53.00 (28.00,81.00)	0.467
PCT (ng/mL)		0.598 (0.130,1.323)	0.236 (0.118,0.938)	0.671
Reported symptoms at presentation				
Time until presentation (d)		4.0 (2.0, 10.0)	5.0 (4.0, 12.0)	0.815
Body temperature (°C)		36.8 (36.5, 38.2)	37.3 (36.6, 39.0)	0.361
Reddening of joints (*n* [%])		13 (35.1)	2 (13.3)	0.116
Joint swelling (*n* [%])		17 (46.0)	2 (13.3)	0.027
Increased skin temperature (*n* [%])		16 (43.2)	9 (60.0)	0.273
Joint pain (*n* [%])		26 (67.6)	12 (80.0)	0.474
Joint involvement (*n* [%])				
Hip joint		25 (67.6)	14 (93.3)	0.078
Knee		10 (27.0)	1 (6.7)	0.145
Ankle		2 (5.4)	0	N/A

*Note*: N/A indicates that no statistical analysis was carried out.

Abbreviations: MRSA, methicillin‐resistant Staphylococcus aureus; MSSA, methicillin‐sensitive Staphylococcus aureus.

## 4 DISCUSSION

Septic arthritis is a common infectious disease in the field of bone and joint in children, and the severity of the disease is related to the type of causative organisms, the length of time between onset and admission, the age of the child, and the effective anti‐infection treatment.^1^ Septic arthritis in children is highly destructive and if not treated in time, the joints can be rapidly destroyed, leading to growth and developmental abnormalities, joint dysfunction, and deformities, with a lethality rate of up to 11%.^5^ The after‐effects can affect the affected child's ability to perform daily activities such as walking, running, and jumping. Prolonged physical discomfort and pain may lead to emotional problems such as anxiety and depression, affecting mental health and placing a heavy financial and psychological burden on families. Therefore, early diagnosis and effective anti‐infective treatment are crucial to the prognosis of the disease. However, it is difficult to accurately identify the pathogenic bacteria in the early stage of infection in clinical practice; thus, it is crucial to accurately determine the pathogenic bacteria as early as possible in combination with different clinical manifestations and laboratory results.

In this study, pathogenic bacteria were isolated from 77 of the 171 patients, with a positive culture rate of 45.0% and a culture‐negative rate of 55.0%, which is in line with previous reports.^6^, ^7^ The reason for the high culture‐negative rate might be related to the low number of pathogenic bacteria.^7^, ^8^ In addition, *Kingella kingae* (*Kk*) has been recognized as an important pathogen for osteoarticular infections in children in Europe.^9^, ^10^, ^11^ However, it is difficult to culture *Kk* on solid media,^12^ and it has been suggested that inoculation of synovial fluid into blood culture bottles ^13^ or the use of polymerase chain reaction (PCR) methods can be effective to increase the detection rate.^14^ However, the above methods are not a part of the routine experimental culture methods, and the PCR method is expensive, which leads to a low detection rate of *Kk*, which is also one of the reasons for the high culture‐negative rate in septic arthritis.

By comparing the blood/synovial fluid culture‐positive and negative groups, we found that the inflammatory markers WBC, CRP, and PCT, and the body temperature were lower in the culture‐negative group than those in the positive group. Previously, Pääkkönen and Feng et al.^15^, ^16^ noted that blood/synovial fluid culture‐negative patients had lower CRP levels than culture‐positive patients, and Spyridakis et al.^7^ found that the blood/synovial fluid culture‐positive and culture‐negative patient groups were similar in symptom presentation on admission; however, the CRP and ESR levels were significantly lower in culture‐negative patients than those in culture‐positive patients. In our study, patients in the culture‐negative group showed more mild symptoms compared with the above‐mentioned reports.

In culture‐positive patients, we found that the main pathogens causing septic arthritis were Gram‐positive bacteria, with *Staphylococcus aureus* dominating, similar to previous studies.^17^ The percentage of SA cases caused by Gram‐negative bacteria was less than the 10%–20%^18^ reported previously. In recent years, because of the widespread use of antibiotics, the resistance of pathogenic bacteria in bone and joint infections has also increased. MRSA is a strain of *Staphylococcus aureus* containing the *mecA* gene or with benzocillin minimum inhibitory concentration (MIC) ≥ 2 μg/mL, and is resistant to all β‐lactam antibiotics currently on the market.^19^ MRSA accounted for 15 (19.5%) of the 52 cases of *S. aureus* in this study, and we found that patients in the MRSA group had a significantly lower rate of joint swelling than those in the MSSA group. This might be attributed to the fact that 14 out of the 15 (93.3%) patients in the MRSA group had hip joint involvement, and when deep joints such as the hip or sacroiliac joints were infected, localized swelling of the joints was not obvious.^20^ In addition, there was no statistically significant difference between the two groups in terms of demographics, first‐time laboratory inflammatory markers, and reported symptoms at presentation, which is in line with the findings of Lin et al.^21^ This result suggested that MSSA and MRSA infections are similar to each other in terms of clinical manifestations and laboratory tests, and MRSA has been reported to have a high rate of resistance to erythromycin and clindamycin, and is sensitive to all vancomycin and linezolid, which can be used as an anti‐infective treatment of choice.^22^

*Streptococcus pneumoniae* is a less frequent cause of SA, and a European prospective study found that only 6.5% of pediatric osteoarticular infections were caused by *Streptococcus pneumoniae*.^23^ Cohen et al.^1^ also found that only 1.6% of patients were infected by *Streptococcus pneumoniae* in a retrospective study. In our study, a two‐by‐two comparative analysis of the *Staphylococcus aureus*, *Streptococcus pyogenes*, and *Streptococcus pneumoniae* groups revealed that the *Streptococcus pneumoniae* group, although small, had a distinctive clinical feature; that is, its median age was 0.69 years, which was significantly lower than that of the *Staphylococcus aureus* group (7 years) and that of the *Streptococcus pyogenes* group (7.75 years).

The present results suggested firstly that *Staphylococcus aureus* and *Streptococcus pyogenes* infections are more likely to be detected at the pre‐ and post‐school age stages. Ø R Riise et al.^24^ reported that patients with childhood septic arthritis caused by *Streptococcus pyogenes* were concentrated in 8–11 years of age, and there are also two case reports suggesting that the age of patients with arthritis caused by *Streptococcus pyogenes* is also between 7 and 8 years of age.^25^, ^26^ Secondly, *Streptococcus pneumoniae* infections are more likely to be detected at the infant stage. Ross reviewed 177 cases of septic arthritis caused by *Streptococcus pneumoniae* reported in the English‐language literature since 1965, as well as 13 patients admitted to the institution, and showed that 62 of the 80 pediatric patients were younger than 2 years of age.^27^ Ispahani et al.^28^ reviewed seven cases of children with septic arthritis caused by *Streptococcus pneumoniae* between 1985 and 1998, six of which were younger than 2 years old. Sánchez Granados^29^ reviewed five patients with septic arthritis caused by *Streptococcus pneumoniae* from 1986 to 2000, four of whom were younger than 15 months of age. Barbeito‐Castiñeiras et al.^30^ Reviewed cases of septic arthritis from 2005 to 2014, where 3 of 77 pediatric patients were affected by *Streptococcus pneumoniae*, 2 of whom were younger than 15 months old. There were also three case reports of septic arthritis caused by *Streptococcus pneumoniae* in which the patients were aged 5, 9, and 10 months, respectively.^31^, ^32^, ^33^ In addition, the *Streptococcus pneumoniae* group was characterized by a longer onset‐admission time, which may be related to the fact that the patients in this group were so young that they were not yet able to communicate or describe their pain and discomfort, so that it took a long time from the onset of the disease to the admission of the patients to the hospital for the symptoms to become typical. This suggests that parents of infants who show a period of refusal to eat, elevated temperature, localized swelling, decreased active joint activity, and passive activity performance should be alert to the possibility of SA.^34^

*Streptococcus pyogenes* can produce a variety of toxins and enzymes, and other pathogenic factors, causing sepsis, necrotizing fasciitis, toxic shock, and other serious invasive infections, representing the most pathogenic type of *Streptococcus*.^35^ In this study, the WBC and PCT levels in the group with *Streptococcus pyogenes* infection were significantly higher than those in the group with *Staphylococcus aureus* and *Streptococcus pneumoniae* infection, and the patients infected with *Streptococcus pyogenes* in this study had a shorter time until presentation. Moreover, it was also found through descriptive statistics that the body temperature and CRP of the *Streptococcus pyogenes* group were also higher than those of the *Staphylococcus aureus* and *Streptococcus pneumoniae* groups. The results of this study show that patients infected with *Streptococcus pyogenes* have an acute condition with severe symptoms and high inflammatory indexes, consistent with Yu et al. report^25^ showing the strong pathogenicity of *Streptococcus pyogenes*.

In summary, patients with SA caused by *Streptococcus pneumoniae* were mostly infants and had a longer time until presentation, whereas patients with *Staphylococcus aureus* and *Streptococcus pyogenes* were mostly school‐age children, and *Streptococcus pyogenes* was more pathogenic than *Staphylococcus* aureus and *Streptococcus pneumoniae*. In addition, another published article of the group analyzed the drug resistance of the pathogenic bacteria in these 171 cases of children with septic arthritis, and it was reported that the results concluded that among the top three pathogenic bacteria, the resistance rate of *S. aureus* to benzoxacillin, amoxicillin/clavulanic acid, cefaclor, and flucloxacillin was <30%, the resistance rate of *Streptococcus pyogenes* to penicillin was 0%, and the resistance rate of *Streptococcus pneumoniae* to penicillin, ceftriaxone resistance rate of 20%, suggesting that the clinic can refer to this result to select antibiotics.^36^

## 5 LIMITATIONS

There are some limitations to this study. Firstly, this was a single‐centered retrospective study with a relatively small sample size and a large time span. Secondly, current PCR and 16S ribosomal RNA or DNA amplification assays are now favorable for improving the culture of picky pathogens^37^, ^38^; macrogenomics is favorable for improving the sensitivity and detection of cultures and is less affected by the previous use of antibiotics.^39^ However, only routine laboratory culture methods were used in this study, and unrecognized *S. aureus* or other pathogenic bacteria may be present in culture‐negative cases; new culture methods will be used in the future. Finally, in this paper, only the clinical characteristics of the groups were analyzed, and the underlying mechanisms of pathogenesis and the prognosis of the patients were not studied, which requires further in‐depth research.

## 6 CONCLUSION

In this study, we reviewed the medical records of children with SA in our hospital in the past 11 years, and analyzed their medical history, clinical manifestations, laboratory tests, and microbiological findings. The results showed that the common pathogenic bacteria that cause SA in children have their own specific infectious characteristics, and clinical manifestations. These clinical manifestations may be useful in guiding treatment in pediatric population.

## AUTHOR CONTRIBUTIONS

The research work is attributed to the Department of Orthopedics, Children's Hospital of Chongqing Medical University. Ziyu Li conceived and designed the study, collected and organized the data, and wrote the article; GeFang Li led the study and participated in data collection and article editing; Bo He provided expertise in the field of osteoarthritic infections and participated in the revision of the article; Jun Wu provided expertise in the field of osteoarthritic infections and participated in the revision of the article; and Qun Zhang participated in the design of the study and provided expert guidance in the field of laboratory science.

## CONFLICT OF INTEREST STATEMENT

The authors declare that the research was conducted in the absence of any commercial or financial relationships that could be construed as a potential conflict of interest.

## ETHICS STATEMENT

This study was a retrospective analysis, involving only case analysis and no human or animal experiments, and was reviewed by the Ethics Committee of Children's Hospital of Chongqing Medical University, Approval No.: 2022‐343.

## Data Availability

The data that support the findings of this study are available from the corresponding author upon reasonable request.
